# Explainable light-weight deep learning pipeline for improved drought stress identification

**DOI:** 10.3389/fpls.2024.1476130

**Published:** 2024-11-28

**Authors:** Aswini Kumar Patra, Lingaraj Sahoo

**Affiliations:** ^1^ Department of Computer Science and Engineering, North Eastern Regional Institute of Science and Technology (NERIST), Itanagar, India; ^2^ Department of Bio-Science and Bio-Engineering, Indian Institute of Technology (IIT) Guwahati, Guwahati, Assam, India

**Keywords:** stress phenotyping, drought stress, machine learning, deep learning, transfer learning, convolutional neural network, explainable machine learning

## Abstract

**Introduction:**

Early identification of drought stress in crops is vital for implementing effective mitigation measures and reducing yield loss. Non-invasive imaging techniques hold immense potential by capturing subtle physiological changes in plants under water deficit. Sensor-based imaging data serves as a rich source of information for machine learning and deep learning algorithms, facilitating further analysis that aims to identify drought stress. While these approaches yield favorable results, real-time field applications require algorithms specifically designed for the complexities of natural agricultural conditions.

**Methods:**

Our work proposes a novel deep learning framework for classifying drought stress in potato crops captured by unmanned aerial vehicles (UAV) in natural settings. The novelty lies in the synergistic combination of a pre-trained network with carefully designed custom layers. This architecture leverages the pre-trained network’s feature extraction capabilities while the custom layers enable targeted dimensionality reduction and enhanced regularization, ultimately leading to improved performance. A key innovation of our work is the integration of gradient-based visualization inspired by Gradient-Class Activation Mapping (Grad-CAM), an explainability technique. This visualization approach sheds light on the internal workings of the deep learning model, often regarded as a ”black box”. By revealing the model’s focus areas within the images, it enhances interpretability and fosters trust in the model’s decision-making process.

**Results and discussion:**

Our proposed framework achieves superior performance, particularly with the DenseNet121 pre-trained network, reaching a precision of 97% to identify the stressed class with an overall accuracy of 91%. Comparative analysis of existing state-of-the-art object detection algorithms reveals the superiority of our approach in achieving higher precision and accuracy. Thus, our explainable deep learning framework offers a powerful approach to drought stress identification with high accuracy and actionable insights.

## 1 Introduction

Abiotic stress adversely affects the development, yield, and quality of the products (de Medeiros et al., 2023). Among various abiotic stresses, soil water deficit or drought stress has the strongest impact on plant health as well as soil biota as drought aggravates other stresses like salinity, heat stress, nutritional deficiency, and pathogen attack which cause further damage to plants (Ahluwalia et al., 2021). Hence, it is essential to detect drought stress at a point where its impacts can be mitigated through prompt irrigation, maximizing the crop’s yield potential. However, the complex nature of drought stress inducing a range of physiological and biochemical responses in plants, operating at both cellular and whole-organism levels (Farooq et al., 2009), make this task increasingly challenging. These responses have been closely associated with specific wavelengths of light that crops reflect and absorb within the visible and near-infra-red (NIR) spectrums (Tucker, 1979). Consequently, various imaging techniques have demonstrated their utility in stress phenotyping (Al-Tamimi et al., 2022). Imaging techniques offer a non-invasive and non-destructive means of identifying plant stress, utilizing a range of methods, including red-blue-green (RGB) imagery (Zubler and Yoon, 2020), thermal imaging (Pineda et al., 2021), fluorescence imaging (Legendre et al., 2021), multi-spectral, and hyper-spectral imaging (Sarić et al., 2022) for stress assessment.

The recent progress in computer vision, particularly artificial intelligence(AI)-driven techniques, including machine learning (ML) and image processing have been extremely useful in detecting and identifying various forms of biotic and abiotic stresses through the utilization of digital image datasets (Li et al., 2020; Gill et al., 2022). While ML models (Singh et al., 2016) have shown considerable success in recognizing plant stress, the manual feature extraction process is constrained by the inability to generalize to diverse tasks, hindering automation and rendering the developed ML model unsuitable for real-time field implementation. In contrast, deep learning (DL), a subset of ML, streamlines the learning process by eliminating the need for manual feature extraction (Singh et al., 2018). Through various convolution layers, DL, particularly convolutional neural networks (CNN), achieves hierarchical feature extraction by automatically extracting valuable information from images. This breakthrough in image classification has been extensively applied to identify and categorize various forms of biotic and abiotic stresses using digital image datasets (Jiang and Li, 2020).

Considerable advancement has been made in drought stress phenotyping through the recent application of ML and DL methods. Zhuang et al. (2017) employed a methodology involving segmentation, followed by the extraction of color and texture features. They implemented a supervised learning method, gradient boosting decision tree (GBDT), to identify water stress in maize. A subsequent study utilizing the same RGB dataset revealed that a deep convolutional neural network (DCNN) outperformed GBDT in terms of performance (An et al., 2019). Detection of water stress in groundnut canopies through hyperspectral imaging is employed, involving various phases for assessing image quality, denoising, and band selection, and eventually classifying using Support Vector Machine (SVM), Random Forest (RF), and Extreme Gradient Boosting (XGBoost/XGB) (Sankararao et al., 2023). Ramos-Giraldo et al. (2020) conducted the classification of four levels of drought severity in soybean color images using a transfer learning technique and a pre-trained model based on DenseNet-121. Azimi et al. (2021) generated a dataset under controlled conditions for chickpea crops, aiming to identify water stress at three stages: control, young seedling, and pre-flowering. They employed a CNN and long short-term memory (LSTM) combination, where CNN architectures functioned as feature extractors, and LSTM was employed for predicting the water stress category. Chandel et al. (2021) investigated the potential of CNN models based on deep learning techniques for accurately identifying stress and non-stress conditions in water-sensitive crops, including maize, okra, and soybean. Three different CNN models—AlexNet, GoogLeNet, and Inception V3—were utilized to assess their efficacy in identification accuracy, revealing that GoogLeNet outperformed the other models. Gupta et al. (2023) utilizes chlorophyll fluorescence images of wheat canopies, employing a multi-step approach that involves segmentation and feature extraction. Feature extraction is done through correlation-based gray-level co-occurrence matrix (CGLCM) and color features (proportion of pixels in each of the nine selected bands). Subsequently, machine learning classifiers are employed for classification, using tree-based methods, particularly the random forest (RF) and extra trees classifier, demonstrating superior performance. Chen et al. (2022) applied the regression approach to predict the drought tolerance coefficient using SVM, RF, and Partial Least Squares Regression (PLSR) based on hyperspectral images of the tea canopy, the findings revealed that SVM outperformed the other two models. A study by Dao et al. (2021) evaluated the effectiveness of machine learning and deep learning methods in detecting drought stress using both full spectra and first-order derivative spectra, comparing their performance with the traditional use of spectral indices. The findings demonstrated the benefits of employing derivative spectra for identifying changes in the entire spectral curves of stressed vegetation, emphasizing the robustness of deep learning algorithms in capturing this complex change. A dataset comprising RGB images of maize crops was curated by Goyal et al. (2024) and their proposed custom-designed CNN model showcases superior performance compared to five prominent state-of-the-art CNN architectures, namely InceptionV3, Xception, ResNet50, DenseNet121, and EfficientNetB1, in the early detection of drought stress in maize. Butte et al. (2021) generated a dataset comprising aerial images of potato canopies and introduced a DL-based model designed to identify drought stress, leveraging diverse imaging modalities and their combinations.

While DL models often outperform traditional Machine ML methods in plant phenotyping tasks, their “black-box” nature makes it difficult to understand how they arrive at decisions. This lack of interpretability is a growing concern as practitioners seek models to deliver accurate results and justify their decisions. There is a limited number of explainable DL models in plant phenotyping research. Ghosal et al. (2018) built a model that accurately identifies soybean stress from RGB leaf images. This model offered valuable insights by highlighting the visual features crucial for its decisions. While Nagasubramanian et al. (2020) acknowledged the importance of interpretability, their approach lacked a detailed explanation of the methods used. Our work addresses these challenges by introducing a novel DL architecture for drought stress assessment in potatoes using aerial imagery. Even with a smaller dataset, our framework achieves superior results compared to the existing methods. Our work offers three key benefits: 1) A transfer learning-based model that effectively leverages knowledge from larger datasets to address the limitations of smaller potato crop stress datasets, overcoming challenges like over-fitting, 2) A light-weight DL pipeline specifically designed to enhance stress identification in potato crops, 3) Integration of Gradient-based visualization for model explainability, highlighting the image regions most relevant to stress detection.

## 2 Materials and methods

### 2.1 Data set description

The potato crop aerial images utilized in this study have been sourced from a publicly accessible dataset encompassing multiple modalities (Butte et al., 2021). Collected from a field at the Aberdeen Research and Extension Center, University of Idaho, these images serve as valuable resources for training machine learning models dedicated to crop health assessment in precision agriculture applications. Acquired using a Parrot Sequoia multi-spectral camera mounted on a 3DR Solo drone, the dataset features an RGB sensor with a resolution of 4,608 × 3,456 pixels and four monochrome sensors capturing narrow bands of light wavelengths: green (550 nm), red (660 nm), red-edge (735 nm), and near-infrared (790 nm), each with a resolution of 1,280 × 960 pixels. The drone flew over the potato field at a low altitude of 3 meters, with the primary objective of capturing drought stress in Russet Burbank potato plants attributed to premature plant senescence.

**Figure 1:**
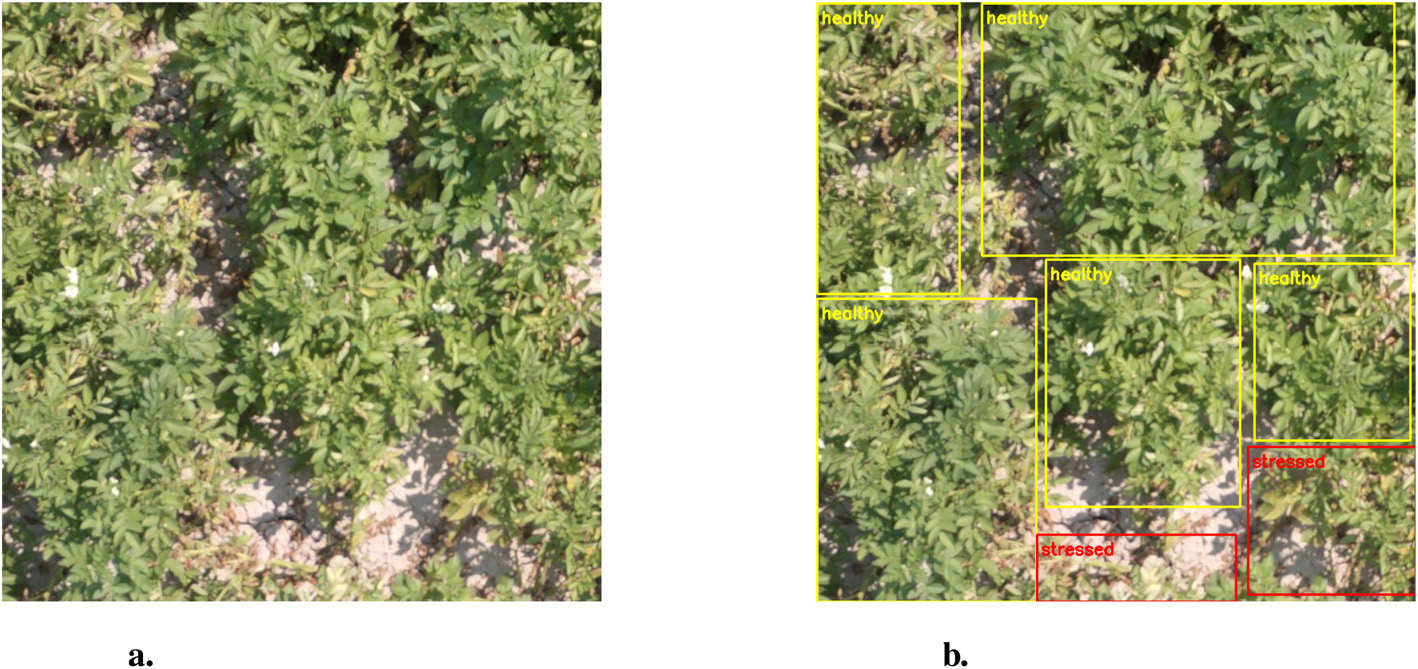
Field images showing **(A)** sample RGB image and **(B)** healthy and stressed plants.

The dataset comprises 360 RGB image patches in JPG format, each sized 750×750 pixels. These patches were obtained from high-resolution aerial images by cropping, rotating, and resizing operations. The dataset is split into a training subset of 300 images and a testing subset of 60. Ground-truth annotations are provided in XML and CSV formats, indicating regions of healthy and stressed plants outlined by rectangular bounding boxes. Annotation was performed manually using the *LabelImg* software. **Figure 1A** shows a sample RGB field image, while **Figure 1B** presents the corresponding annotated regions, with yellow representing healthy areas and red indicating stressed areas. The testing subset is independent of the training subset sourced from different aerial images. The dataset also includes image patches from spectral sensors featuring red, green, red-edge, and near-infrared bands, with a size of 416×416 pixels. However, we are solely utilizing RGB images due to the limitations of the low-resolution monochromatic images.

In this study, we utilized 1500 augmented images generated from the original 300 images using the method devised by Butte et al. (2021). From each augmented image, annotated windows—rectangular bounding boxes with provided coordinates in the data repository—were extracted, resulting in separate “healthy” and “stressed” classes. The final count of images for the “stressed” and “healthy” classes were 11,915 and 8,200, respectively. These images underwent further augmentation during training, as outlined in the proposed model. The evaluation of the model was performed on a specific test set comprising 60 images, from which 401 healthy images and 734 stressed images were extracted using the bounding boxes similar to the process used for the training image set.

### 2.2 Proposed methodology

We present an integrated approach for drought stress classification, featuring a CNN-based pipeline with transfer learning and an interpretability technique to enhance model transparency. This methodology combines data augmentation, transfer learning, and a CNN architecture for robust feature extraction, followed by explainability methods that leverage gradients to provide insights into the model’s decision-making process. The methodology is structured as follows:

#### 2.2.1 Deep learning pipeline with transfer learning

**Figure 2:**
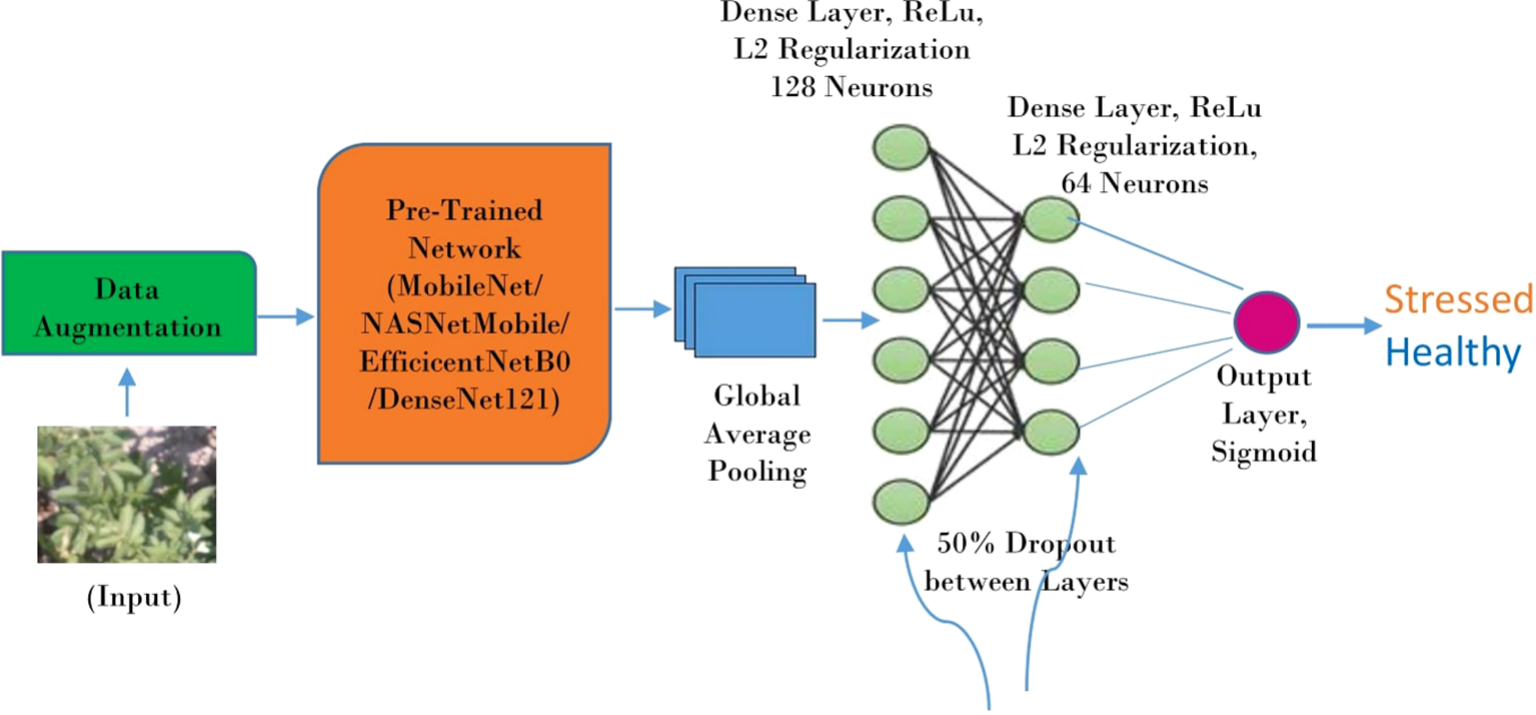
Deep learning framework for drought stress identification.

The proposed framework uses CNN-based architecture with transfer learning to differentiate between drought-stressed and healthy plants. Transfer learning enables the model to start with a pre-trained network, reducing training time and improving accuracy, especially with smaller datasets. The model is divided into three key components: data augmentation, a pre-trained network, and additional layers for final classification. The pipeline is depicted in **Figure 2**.

- **Data Augmentation:** It tackles the challenge of limited training data by artificially expanding the dataset with variations of existing samples. This approach injects variability and improves the model’s generalization ability to unseen data. Transformations like re-scaling, shearing, rotating, shifting, and flipping are applied to create a more diverse training set. This robustness to variations helps the model perform better on real-world data and reduces the risk of over-fitting.
- **Pre-trained Network:** Transfer learning is employed to speed up the training process and improve accuracy by starting with a pre-trained CNN. The pre-trained model serves as the backbone of the architecture, effectively extracting low-level and mid-level features from the images. Networks like EfficientNetB0, MobileNet, DenseNet121, and NASNetMobile, trained on vast and diverse datasets such as ImageNet, are repurposed to recognize drought stress by fine-tuning them for this specific task.
- **Additional Layers:** Two types of layers are applied after the pre-trained architecture: the Global Average Pooling Layer and Dense Layers.
- **Global Average Pooling:** It reduces the dimensionality of spatial data (like feature maps from convolutional layers) into a single feature vector. It achieves this by calculating the average of all elements within each feature map, resulting in one value per feature map.
- **Dense layers:** Two fully connected dense layers are stacked sequentially after global average pooling. These layers perform computations to learn complex relationships between the features extracted by the pre-trained network. Dropout and L2 regularization are applied between each dense layer to prevent over-fitting. Dropout randomly drops a certain percentage of neurons during training, forcing the model to learn from different subsets of features and reducing its reliance on any specific feature. L2 regularization penalizes large weights in the model, discouraging the model from becoming overly complex. Each neuron in the dense layer applies a weighted sum and activation function (ReLU) to determine the probability of an image belonging to a particular class.
- **Output Layer:** This layer uses a sigmoid activation function to generate the final probability scores between 0 and 1, indicating stressed or healthy.

In essence, the model incorporates data augmentation to enrich the training data, takes advantage of a pre-trained network’s feature extraction capabilities, and uses dense layers with regularization and dropout to learn a classification boundary between stressed and healthy images.

#### 2.2.2 Explainability through gradient-based visualization

We integrated a gradient-based explainable approach into our framework to ensure model transparency and to enhance interpretability. It is inspired by Grad-CAM (Selvaraju et al., 2017), a technique that provides visual explanations by highlighting the regions of input images that contribute most to the model’s predictions. While Grad-CAM focuses on class-specific, high-level features, gradient-based visualization emphasizes pixel-level sensitivities, offering a broader perspective on what influences the model’s output. By visualizing the areas most relevant to the model’s decision, the devised explainable approach offers valuable insights into the decision-making process of the deep learning model. The practical application involves taking an input image that the deep learning pipeline can classify as healthy or stressed. According to the trained model, we can then use the identified stressed image to locate the specific areas of the field that are affected by stress. The following steps are involved in the proposed explainable approach, which takes its cue from Grad-CAM.

1. **Forward Pass:** The model output *θ* is computed by performing a forward pass through the deep learning model, represented as:


θ=ϕ(ξ)

where:

-
θ represents the model output.
-
ϕ(·) represents the deep learning model.
-
ξ represents the input image.

2. **Compute Gradients:** The gradients of the model output with respect to the input image are calculated, represented as:


$$\nabla_{\xi }\theta =\frac{\partial \theta }{\partial \xi }$$

where:

-
$\nabla_{\xi }\theta$ represents the gradients of the model output with respect to the input image.
-
$\frac{\partial \theta }{\partial \xi }$ represents the partial derivatives of the model output with respect to the input image.

3. **Gradient Visualization:** The absolute gradients are computed and visualized as a heatmap, represented as:


$$\text{Heatmap}=\text{abs}\left(\nabla_{\xi }\theta \right)$$

where:

- Heatmap represents the heatmap visualization of the gradients.
- abs(·) represents the absolute value function.

4. **Standardization:** The heatmap is optionally standardized by subtracting the mean and dividing by the standard deviation to improve visualization, represented as:


$${\text{Heatmap}}_{\text{std}}=\frac{\text{Heatmap}-\mu }{\sigma }$$

where:

- Heatmap_std_ represents the standardized heatmap.
- *µ* represents the mean of the heatmap.
- *σ* represents the standard deviation of the heatmap.

5. **Plotting:** Finally, the input image and the heatmap are plotted side by side for visualization.

Thus, the explainable approach based on Grad-CAM leverages the strength by analyzing gradients to pinpoint image regions crucial for the model’s decisions, offering valuable insights into what triggers the model’s stress responses.

### 2.3 Evaluation metrics

**Figure 3:**
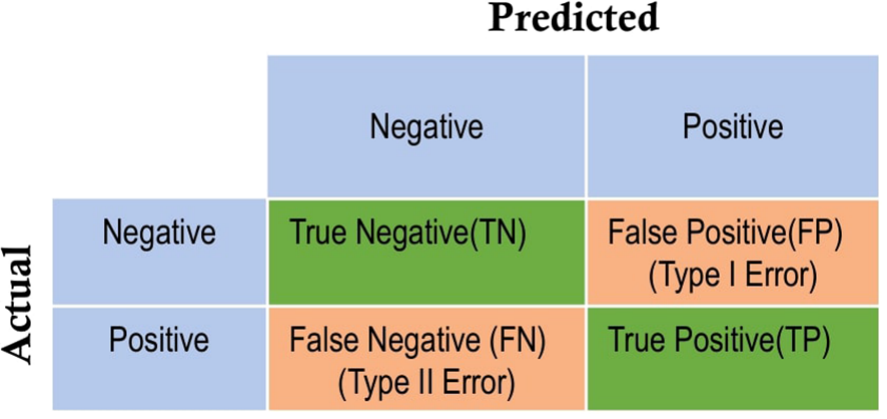
Confusion matrix.

The model’s performance underwent assessment using various evaluation metrics, including accuracy, precision, and recall (sensitivity). These metrics are computed based on the counts of true positives (TP), true negatives (TN), false positives (FP), and false negatives (FN), which collectively form a 2x2 matrix known as the confusion matrix. The format is illustrated in **Figure 3**, where the negative class represents the “healthy” class, and the positive class corresponds to the stressed class.

In this matrix, TP and TN indicate the accurate predictions of water-stressed and healthy potato crops, respectively. FP, termed type 1 error, denotes predictions where the healthy class is inaccurately identified as water-stressed. FN, referred to as type 2 error, represents instances where water-stressed potato plants are incorrectly predicted as healthy. The classification accuracy is a measure of the ratio between correct predictions for stressed and healthy images and the total number of images in the test set. Precision is the ratio of true positives to the sum of true positives and false positives, indicating the proportion of correctly identified positive instances out of all instances predicted as positive. Recall (sensitivity) is the ratio of true positives to the sum of true positives and false negatives, reflecting the model’s ability to correctly identify all positive instances. The formulas for accuracy, precision and recall are given below.


$$\text{Accuracy}=\frac{\text{True Positive}+\text{True Negative}}{\text{Total Population}}$$

$$\text{Precision}=\frac{\text{True Positive}}{\text{True Positive}+\text{False Positive}}$$

$$\text{Recall}=\frac{\text{True Positive}}{\text{True Positive}+\text{False Negative}}$$

### 2.4 Model workflow

**Figure 4:**
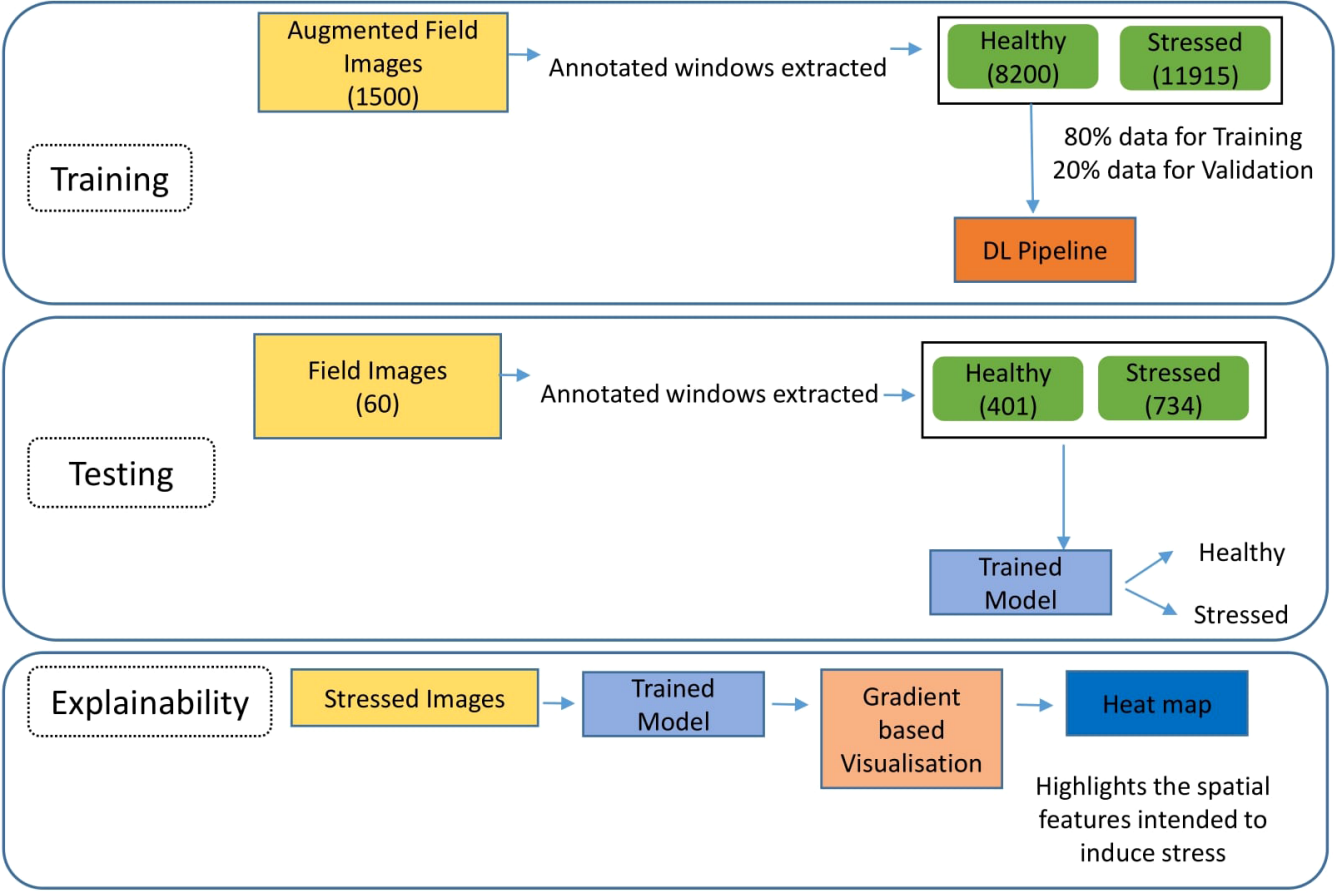
Work-flow of the model.

The proposed pipeline is a comprehensive framework that involves model training, evaluation, and explainability to provide a robust and transparent solution for identifying stressed plants in field images. It is demonstrated in **Figure 4**. The training phase of the pipeline utilizes a dataset of 1500 augmented field images, each annotated with bounding boxes to delineate regions of healthy and stressed plants. These annotated windows were extracted from each augmented image, resulting in separate “healthy” and “stressed” classes with 8,200 and 11,915 images, respectively. The dataset is divided into 80% for training and 20% for validation to prevent over-fitting. In the testing phase, a distinct testing dataset comprising 60 field images are employed to evaluate the model’s performance on unseen data. The evaluation is conducted on a test set of 60 images, with 401 healthy and 734 stressed images extracted using bounding boxes. The model’s performance is assessed using standard evaluation metrics such as accuracy, precision, and recall. Then, to understand the model’s decision-making process, the pipeline incorporates a devised explainable approach based on gradients. It involves using an already identified stressed image as input, leveraging the trained model and gradient-based visualization techniques to generate heatmaps highlighting the areas of the image the model identifies as affected by stress. These heatmaps provide valuable insights into the model’s reasoning and can help identify the visual cues that indicate plant stress.

## 3 Results and discussion

**Figure 5:**
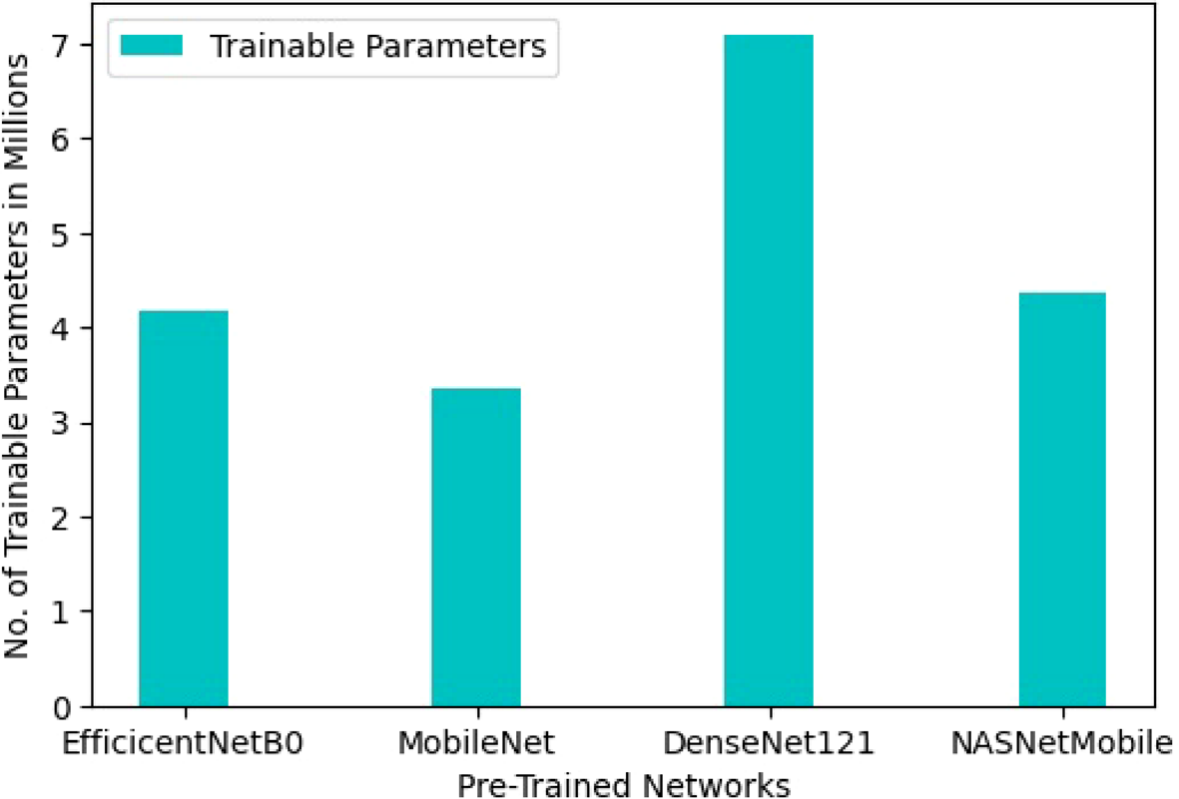
Number of trainable parameters of the model with different pre-trained CNN architectures.

The methodology employed in this study utilizes transfer learning, leveraging knowledge from models trained on the ‘ImageNet’ dataset and adapting it to address drought stress identification. By using pre-trained networks as a foundation rather than starting from scratch, the approach reduces storage requirements and computational demands. This approach results in a light-weight model, with trainable parameters ranging from 3.3 million to 7.36 million across various pre-trained networks, a notable departure from the considerably heavier models typically used in deep learning tasks. Specifically, the trainable parameters for EfficientNetB0, MobileNet, DenseNet121, and NASNetMobile are 4.18 million, 3.35 million, 7.09 million, and 4.37 million, respectively, as depicted in **Figure 5**.

In our deep learning framework, *Python* version 3.8.8 serves as the programming language foundation, while *TensorFlow* and *Keras*, widely recognized and utilized libraries, are employed for model development and training. Additionally, various libraries such as *os*, *pandas*, *numpy*, and *sklearn* were employed to facilitate data manipulation and metric calculations.

In the proposed deep learning framework, the input data undergoes augmentation through various transformations. Four pre-trained networks are systematically evaluated, each serving as a backbone feature extractor. Additional layers are stacked on top of these networks to complement their ability to identify drought stress in images collected from natural settings. The following discussion provides an in-depth analysis of the parameters, the pipeline’s performance based on learning curves and confusion matrices, and the model’s explainability by identifying stressed spatial features in field images. In addition, our approach is compared to previous works based on object detection algorithms, demonstrating that the proposed method outperforms them.

### 3.1 Model parameters

The input dataset is divided into two subsets for training and validating, utilizing a fixed random seed of 42. The *random_state* = 42 parameter ensures re-producibility by setting a specific random seed, guaranteeing consistent results across different runs of the code. Separate generators are created for training, validation, and testing datasets using the *ImageDataGenerator* function from *Keras*. Each generator is configured with specific settings tailored to the respective pre-trained architectures: EfficientNetB0, MobileNet, DenseNet121, and NASNetMobile within the deep learning framework as discussed in section 2.2.1. The target image sizes are set to 224x224 for EfficientNetB0, MobileNet, and DenseNet121, and 299x299 for NASNetMobile. The re-scaling factor, batch size, and class mode are standardized across all architectures, with values of 1/255, 128, and *binary*, respectively. The training generator is also equipped with data augmentation transformations to enhance the dataset’s variability and improve model generalization. Key parameters governing these transformations, including the shear range, rotation range, width shift range, and height shift range, are configured as 0.2, 30, 0.2, and 0.2, respectively. Horizontal and vertical flipping are enabled with boolean values set to *True* for both, while the fill mode is specified as *nearest*.

The proposed custom architecture builds on the pre-trained network by adding several layers. It begins with global average pooling, followed by two dense layers utilizing 128 and 64 neurons, respectively. Each dense layer utilizes ReLU activation for efficient learning, dropout with a 50% rate to prevent over-fitting, and L2 regularization with a weight decay of 0.01 to further enhance robustness during feature extraction. The final layer of the network comprises a single neuron with sigmoid activation, outputting a value between 0 and 1, representing the probability of the input belonging to a specific class. The Adam optimizer is employed for training, starting with an initial learning rate of 0.001. An exponential decay schedule is applied to adjust the learning rate over epochs. This schedule gradually reduces the learning rate after every two epochs with a decay rate 0.9. The chosen loss function is *binary cross-entropy*, which measures the difference between the predicted probabilities and the actual class labels.

A callback function is utilized using *ModelCheckpoint* from *Keras* to save the best-performing version of the model during training. This callback monitors the validation loss and saves the model only when a new minimum validation loss is achieved. After training, the code identifies the epoch with the lowest validation loss and loads the corresponding model weights. These weights are then utilized to evaluate the model’s performance on a separate test dataset. This strategy ensures that the model evaluated on unseen data represents the optimal performance attained during training.

### 3.2 Performance of the model

We investigated four pre-trained networks individually as part of the proposed deep learning pipeline. While EfficientNetB0 and NASNetMobile achieved high training accuracies of 99.38% and 98.47%, respectively, their validation and test accuracies were notably lower, indicating potential weaknesses as evidenced by their loss and accuracy learning curves, which is discussed later in the section. In contrast, MobileNet demonstrated impressive performance with a training accuracy of 99.81%, a validation accuracy of 99.33%, a test accuracy of 88.72%, and a low validation loss of 0.033. Similarly, DenseNet121 showcased robust performance across training, validation, and test sets, achieving a training accuracy of 99.69%, a validation accuracy of 98.86%, and a test accuracy of 90.75%. Overall, DenseNet121 emerged as the best-performing model among those investigated, boasting the highest test accuracy, closely followed by MobileNet. The comparative performance is summarized in **Table 1**. Epochs in training are chosen based on observing the convergence pattern of the model, typically by monitoring performance metrics on a validation dataset. The training continues until the model’s performance on the validation set plateaus or degrade, indicating convergence and preventing over-fitting. The deep learning pipeline was trained with EfficientNetB0, MobileNet, DenseNet121, and NASNetMobile for 30, 60, 60, and 30 epochs, respectively. The optimal performance for each model was achieved at epochs 30, 59, 55, and 28, correspondingly.

**Table 1:** Model performance.

Model	Train Acc	Val Acc	Val Loss	Test Acc	No. Epoch	Best Result Epoch
EfficientNetB0	99.38	74.30	0.5033	74.00	30	30
MobileNet	99.81	99.33	0.0330	88.72	60	59
DenseNet121	99.69	98.86	0.0508	90.75	60	55
NASNetMobile	99.47	59.81	0.6815	64.67	30	28

**Figure 6:**
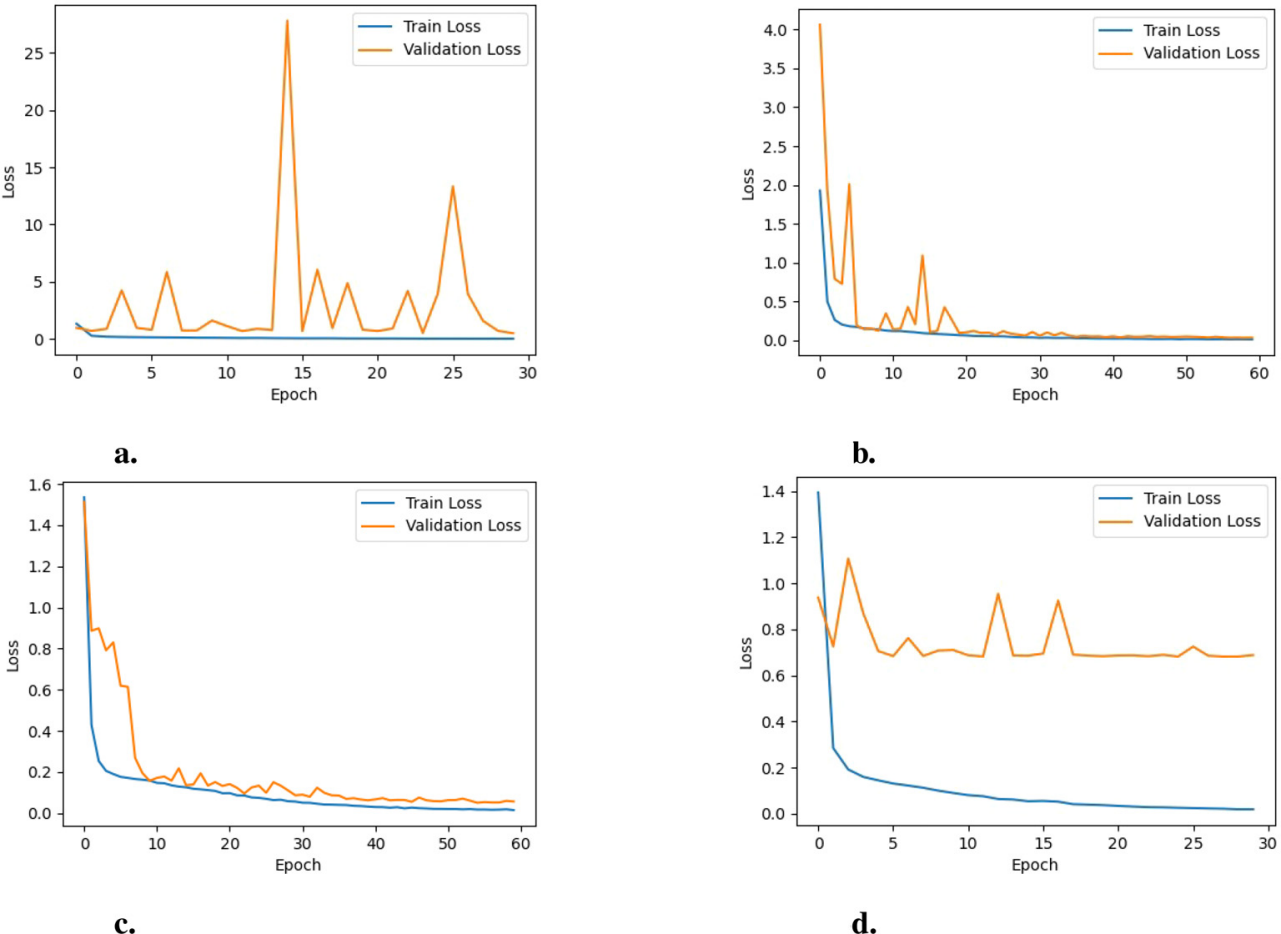
Training loss vs. validation loss of the model for the various PreTrained Networks: **(A)** EfficientNetB0, **(B)** MobileNet, **(C)** DenseNet121 and **(D)** NASNetMobile.

**Figure 7:**
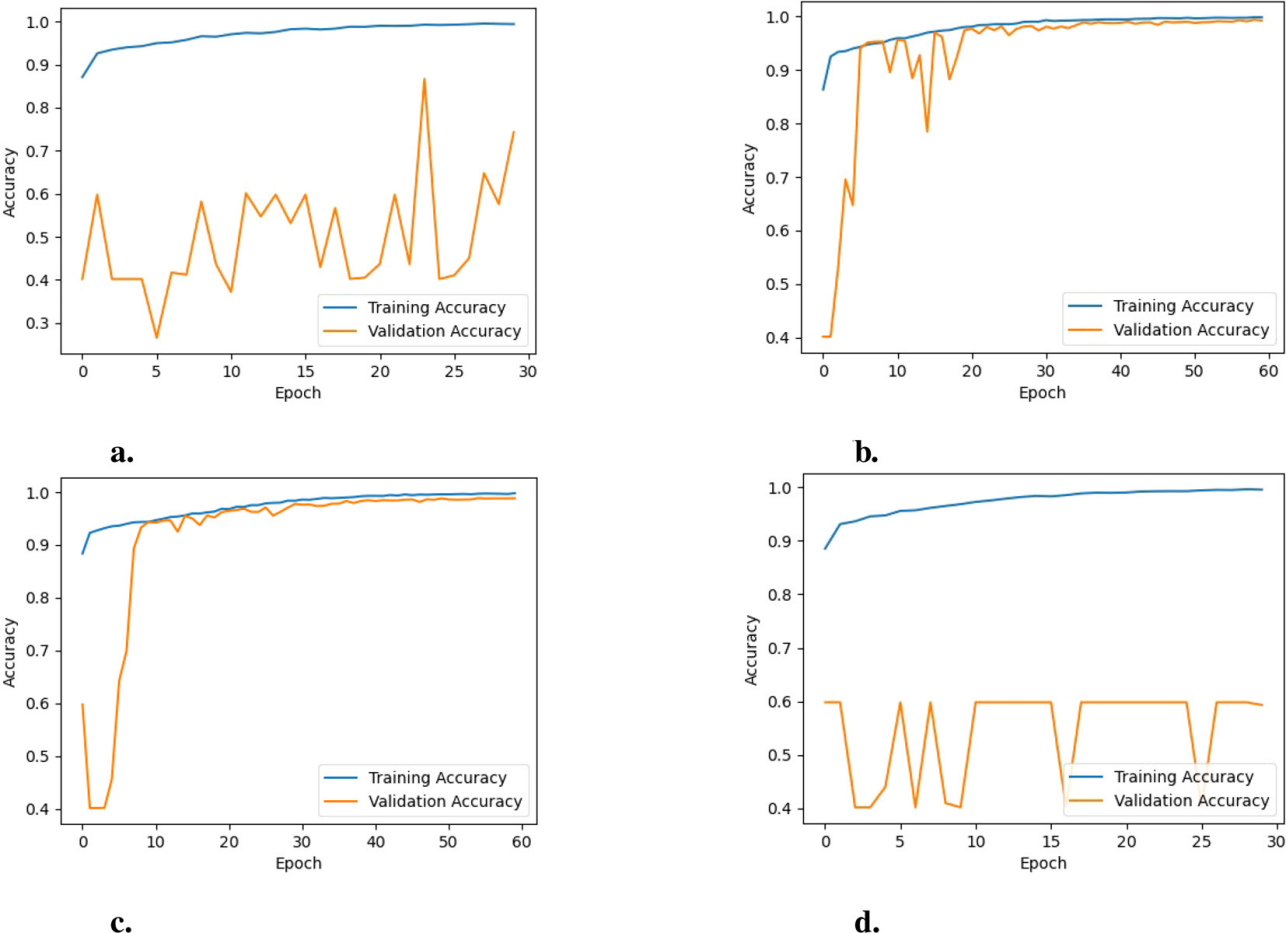
Training vs. validation accuracy of the model for the various PreTrained Networks: **(A)** EfficientNetB0, **(B)** MobileNet, **(C)** DenseNet121, and **(D)** NASNetMobile.

**Analysis of learning curves:** Analyzing the learning curves for training and validation loss and training and validation accuracy offers valuable insights into how the model performs and behaves throughout the training process when employing different pre-trained networks. For EfficientNetB0, as illustrated in **Figure 6A**, the training loss stabilizes at a low value, indicating that the model has learned most of the patterns in the training data and is not finding any substantial new information. On the other hand, the fluctuating validation loss indicates that the model’s performance on unseen data (the validation set) is inconsistent, suggesting potential over-fitting or instability during training. Furthermore, the training accuracy remains consistently high, as shown in **Figure 7A**. In contrast the validation accuracy fluctuates more, implying that the model performs well on the training data but struggles to generalize effectively to unseen data. Additionally, the noticeable gap between the training and validation accuracy further suggests over-fitting, where the model becomes too specialized to the training data and fails to generalize well to new data.

For NASNetMobile, as depicted in **Figure 6D**, the learning curves for training and validation loss reveal evidence of over-fitting, given the considerable gap between the two curves. Regarding training and validation accuracy learning curves, a similar pattern is observed, as shown in **Figure 7D**. This suggests that while these models perform well on the training data, their performance on unseen validation data is substantially lower.

For DenseNet121, the trends observed in the loss graphs indicate that the model is effectively learning from the data. Both training and validation loss curves (i.e., **Figure 6C**) demonstrate a consistent decrease over time. While there is an initial gap between the training and validation loss curves, this gap gradually diminishes as the training progresses. This narrowing gap suggests the model is improving its generalization ability to unseen data. Additionally, the validation accuracy steadily increases throughout the training process and remains closely aligned with the training accuracy(i.e., **Figure 7C**), indicating the model’s positive performance on both training and validation sets. The performance of MobileNet exhibits a similar trend, where the loss graphs indicate effective learning by the model. Both training and validation loss curves (i.e., **Figure 6B**) depict a consistent decrease over time. Nonetheless, a noticeable gap persists between the training and validation loss curves, suggesting a potential for over-fitting, although not severe, given the concurrent increase in validation accuracy (i.e., **Figure 7B**). This indicates that the model is still able to generalize well to unseen data, despite the observed gap between the loss curves.

**Figure 8:**
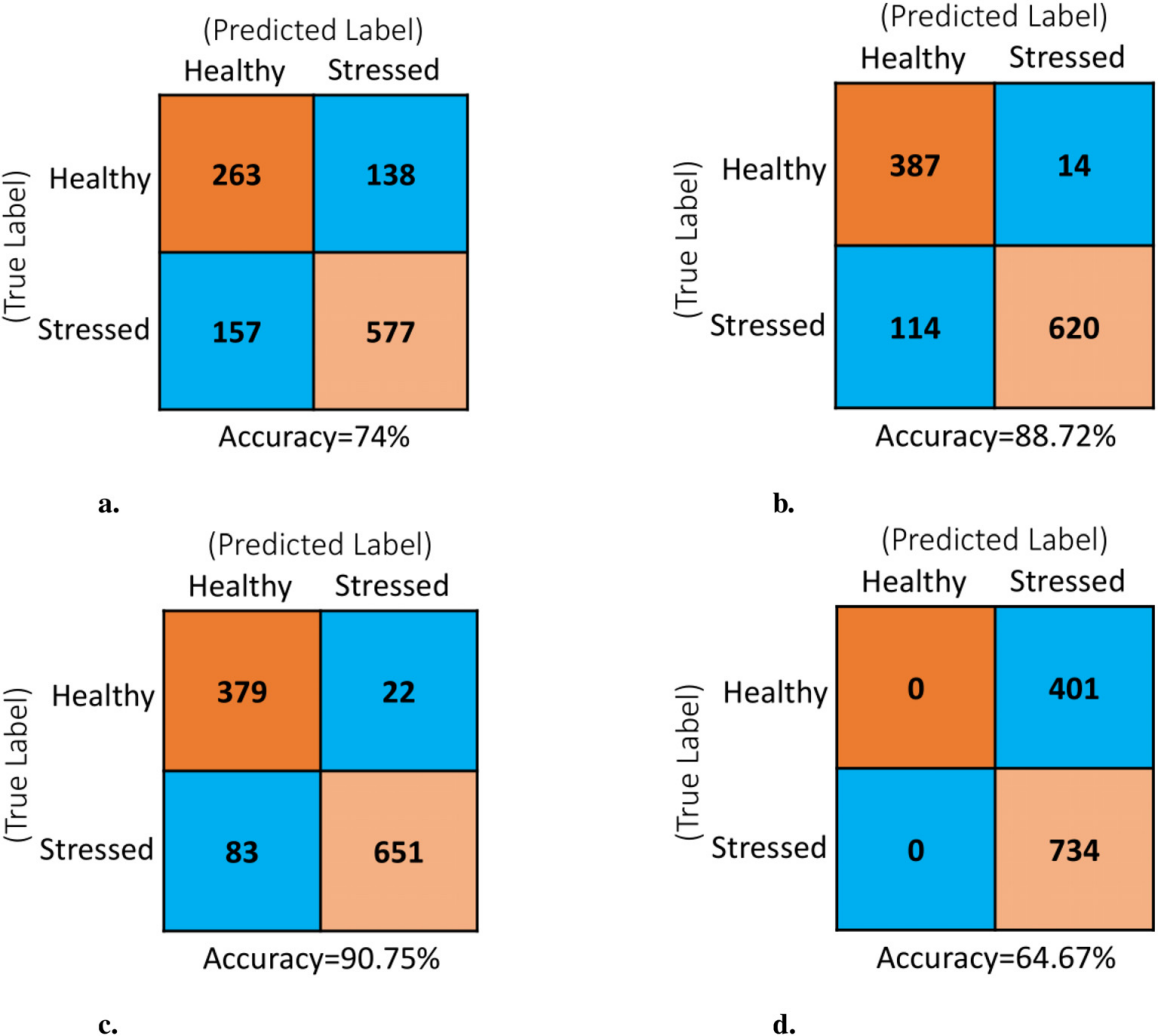
Confusion matrix of the model for the various PreTrained networks with test data set comprising of 1135 images: **(A)** EfficientNetB0, **(B)** MobileNet, **(C)** DenseNet121 and **(D)** NASNetMobile.

**Analysis of Confusion Matrices:** The analysis of confusion matrices is essential to provide deeper insight into the model’s performance beyond accuracy. The deep learning pipeline utilizing various pretrained CNN models were evaluated on an independent test set of 1,135 images that were not part of the training process. The model generated the confusion matrices shown in **Figure 8** using these already trained networks. The values within each confusion matrix were arranged according to the layout shown in **Figure 3**. Following the similar pattern observed in the previous learning curves, the EfficientNetB0 and NASNetMobile showed the poorest performance on the test dataset. For EfficientNetB0, analysis of the confusion matrix (**Figure 8A**) reveals a total of 295 misclassified instances out of 1135 predictions, comprised of 138 false positives (FP) and 157 false negatives (FN). This results in a misclassification rate of 26%. In contrast, the confusion matrix for NASNetMobile (**Figure 8D**) indicates a strange behavior where the model correctly identifies all stressed images but fails to recognize any healthy ones. In the case of EfficientNetB0, the higher misclassification rate suggests suboptimal performance across both classes. Conversely, NASNetMobile’s performance is characterized by a notable bias towards the “stressed” class, resulting in a complete oversight of the “healthy” class. Both scenarios are deemed undesirable, rendering the models ineffective for their intended purpose. On the other hand, both MobileNet and DenseNet121 achieve very low misclassification rates between healthy and stressed classes, as shown by the minimal Type I and Type II errors in their respective confusion matrices (**Figures 8B, C**). This translates to high overall accuracies of 88.72% for MobileNet and 90.75% for DenseNet121.

DenseNet121 stands out as the top-performing backbone in the proposed deep learning pipeline, strengthened by data augmentation and additional layers. Analysis of both learning curves and confusion matrices shows that it generalizes better on unseen data and distinguishes healthy and stressed classes more effectively than EfficientNetB0, MobileNet, and NASNetMobile.

### 3.3 Explaining the model

We employ a method to generate visual explanations for decisions made by the proposed deep learning pipeline, enhancing its transparency. We utilize the DenseNet121 pre-trained network in our pipeline due to its superior performance compared to other networks. The explanations are derived from analyzing gradients at two distinct stages of the pipeline, resulting in two scenarios for investigation:

- Scenario 1: Gradients are considered at the last dense layer.
- Scenario 2: Gradients are considered at the last convolutional layer of DenseNet121.

**Figure 9:**
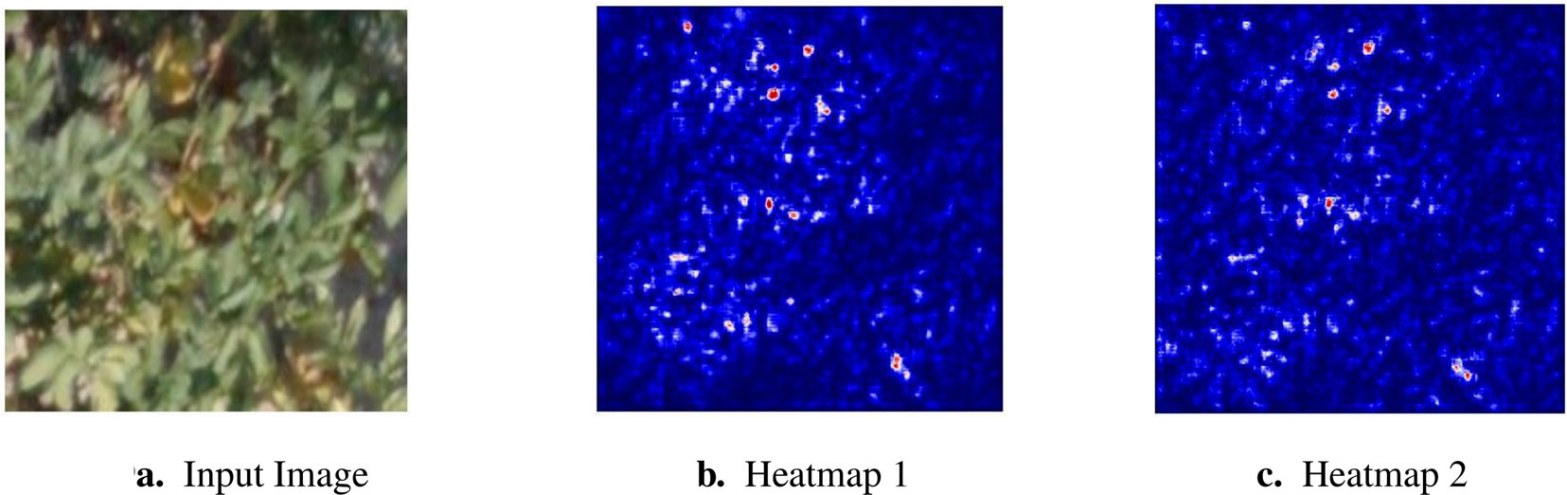
Explaining the deep learning model using gradient-based visualization. **(A)** Input Image **(B)** Heatmap 1. **(C)** Heatmap 2.

These gradients are used to generate a coarse localization map for a specific target concept, such as drought stress. This map highlights key regions within the image that contribute greatly to predicting the concept. Analyzing an RGB image for drought stress involves examining various visual cues and patterns indicative of plant stress. In such images, areas of interest often exhibit discoloration, wilting, or reduced foliage density compared to healthy regions. The color spectrum may shift towards yellow or brown, signifying decreased chlorophyll content and photosynthetic activity. Additionally, leaf curling or necrotic spots may be visible, indicating water scarcity and cellular damage. The explainability process begins with pre-processing the drought-stressed image by resizing it to match the model’s input dimensions and normalizing the pixel values to ensure consistent data representation. After pre-processing, the image is fed into the trained deep learning model. We used the model weights from the 55th epoch, as they provided the best performance in terms of classification accuracy, precision, and recall. Gradients are then calculated using *GradientTape*, a *TensorFlow* component that facilitates automatic differentiation. These gradients are subsequently used to generate a heatmap that effectively highlights the critical regions within the input image that contribute to the prediction of drought stress. The entire process is summarized in the **Algorithm 1**. The generated heatmap overlays the original image, highlighting areas where the model places greater importance in its decision-making process. The original image, along with the heatmaps for both Scenario 1 and Scenario 2, are shown in **Figures 9A–C**, respectively. The *seismic* colormap is used for heatmap visualization, where red shades highlight areas of high importance, blue indicates regions of low importance, and white represents the neutral point.

Algorithm 1 **Visualize image regions associated with stress.**

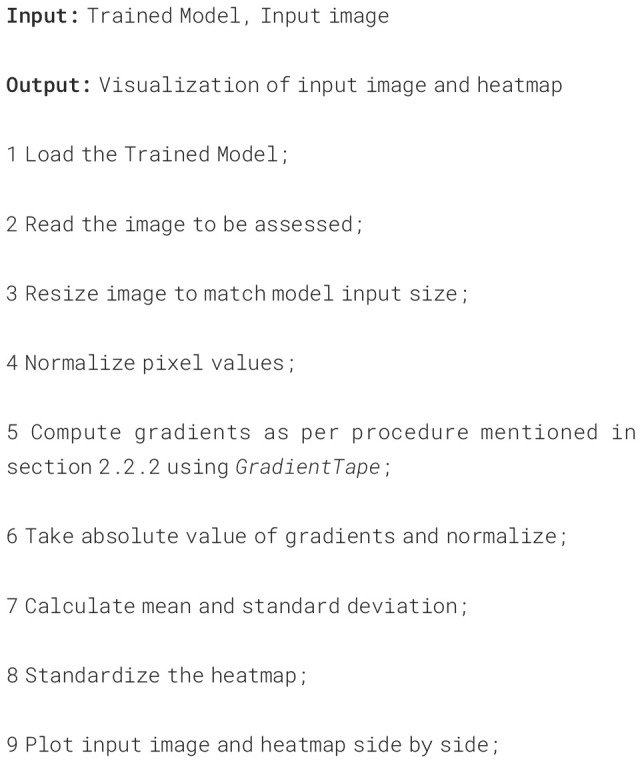

The sensitivity analysis is conducted to evaluate the robustness of the model against noise and small changes in the input in both scenarios. Specifically, it assesses how a trained model’s predictions are influenced by perturbations in input images through the addition of Gaussian noise. This analysis involves introducing Gaussian noise to 148 images, which represent 20% of the drought-stressed images from the test set, using a variance of 0.01. Sensitivity is measured by calculating the absolute difference in prediction scores between the original and perturbed images, providing a numerical sensitivity score that reflects the model’s resilience to slight variations in input data. To summarize the findings, key statistics are computed, including the average sensitivity, median sensitivity, and standard deviation of the sensitivity scores across all tested images. Furthermore, the distribution of these sensitivity scores is visualized using a histogram, with the score plotted against frequency and a bin size of 10.

In Scenario 1, the median sensitivity score is 1.65, close to the average sensitivity of 1.73, indicating a consistent response to noise across various inputs. These findings suggests that the model’s predictions remain relatively stable and predictable, with minimal variation in how noise affects the different inputs. The standard deviation in Scenario 1 is 0.71, further emphasizing the model’s consistency in handling noise, as the spread of sensitivity scores is narrow, and there are fewer outliers. In contrast, Scenario 2 exhibits a higher median sensitivity of 2.32. Still, it is notably lower than the average sensitivity of 3.01, indicating that while many inputs show moderate sensitivity to noise, a few outliers with much higher sensitivity skew the average upward. These observations suggests that the model’s response to noise is less consistent in Scenario 2, as the presence of outliers introduces greater variability. The standard deviation 2.48 in Scenario 2 reflects this wider spread of sensitivity scores, highlighting the model’s reduced robustness when gradients are taken from the convolutional layer. The greater variability indicates that some images are more affected by noise than others, making the model’s behavior less predictable in this scenario.

**Figure 10:**
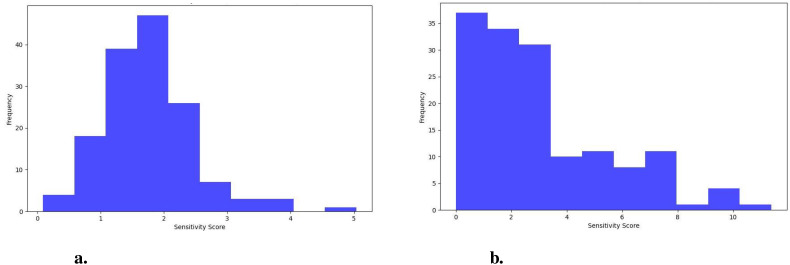
Distribution of sensitivity scores **(A)** Scenario 1 and **(B)** Scenario 2.

The distribution of sensitivity scores for Scenario 1 (**Figure 10A**) and Scenario 2 (**Figure 10B**) further supports these findings. In Scenario 1, the distribution is concentrated around lower sensitivity scores, with most images showing sensitivity scores below 2. This pattern indicates that the model is generally less sensitive to noise, with fewer outliers, reflecting better stability and consistency across inputs. In contrast, Scenario 2 exhibits a wider range of sensitivity scores, from 0 to 10, indicating much higher variability. Some images show very high sensitivity scores, reaching up to 10, suggesting that some of the inputs are more affected by noise perturbations than others. Thus, Scenario 1 demonstrates better stability and interpretability, while Scenario 2 is more prone to noise and shows greater variability in its responses.

In summary, gradient-based visualization of drought-specific spatial features helps bridge the gap between a CNN model’s ‘black box’ nature and human understanding. It empowers agricultural practitioners to interpret the model’s reasoning and make informed decisions about plant health based on visual cues and analysis.

### 3.4 Performance comparison with object detection methodologies

We compare our proposed classifier, which incorporates gradient-based explainability, with the object detection algorithms implemented in a previous work (Butte et al., 2021). This comparison is particularly insightful because the localization aspect of object detection models aligns with our proposed approach, which also focuses on pinpointing stress areas in crops. Both systems are designed to identify and distinguish between two classes (stressed and healthy) using the same dataset and bounding boxes.

**Figure 11:**
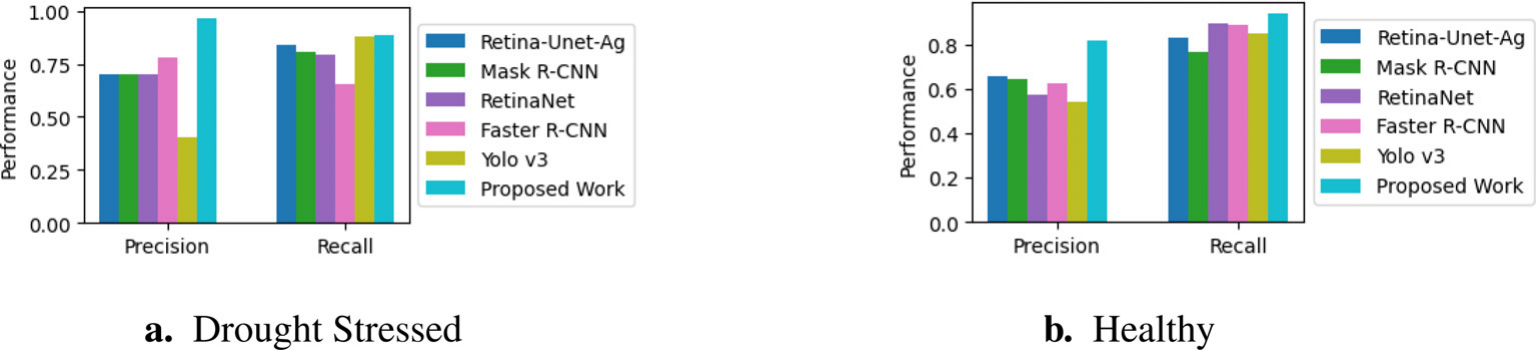
Comparison of precision and recall metrics across various models. **(A)**. Drought Stressed **(B)**. Healthy.

The evaluation is based on precision and recall metrics to measure each model’s effectiveness in accurately detecting instances of the target classes. Higher precision and recall indicate superior performance in classifying stressed and healthy conditions. **Table 2** presents the performance metrics of our proposed model and compares them with those of models reported by (Butte et al., 2021). Our proposed pipeline, based on DenseNet121 notably outperforms the other models. It achieves the highest precision for both stressed (0.967) and healthy (0.820) instances, along with the best recall for stressed instances (0.887). These results demonstrate its ability to accurately identify stressed conditions while maintaining high precision and minimizing false positives. In contrast, while Yolo v3 shows competitive recall for stressed plants (0.882), its low precision (0.407) indicates that it frequently misclassifies healthy plants as stressed. The performance comparison is further visualized in the histogram shown in **Figure 11**. This result shows that our method provides the reliable and accurate classification of stressed and healthy conditions compared to traditional object detection models.

**Table 2:** Performance of the models with the RGB images.

Model	Stressed		Healthy	
	Precision	Recall	Precision	Recall
Retina-Unet-Ag	0.702	0.841	0.659	0.832
Mask R-CNN	0.700	0.809	0.644	0.769
RetinaNet	0.698	0.795	0.578	0.899
Faster R-CNN	0.781	0.654	0.630	0.891
Yolo v3	0.407	0.882	0.541	0.855
Proposed Pipeline (with DenseNet121)	0.967	0.887	0.820	0.945

Moreover, the proposed classifier with explainability offers a better alternative to traditional object detection algorithms when interpretability and high precision in identifying stressed plants are prioritized. The visual insights provided by the explainable approach enhance model transparency by highlighting critical regions used in the decision-making process. Such insights are especially useful for applications where understanding the model’s reasoning is crucial, such as early stress detection in agriculture. On the other hand, traditional object detection algorithms may be more appropriate for tasks requiring precise object localization and real-time performance. Therefore, choosing our classifier and object detection models depends on specific application requirements and priorities.

Our work advances non-invasive imaging techniques for crop monitoring by offering an interpretable, high-precision classifier that supports early stress detection. This advancement greatly aid decision-making in agriculture, ultimately contributing to better crop management practices.

## 4 Conclusion

This study confirms the effectiveness of a deep learning pipeline, specifically utilizing DenseNet121 as the backbone, along with a data augmentation procedure and custom layers, to detect drought stress in potato crops with high accuracy. The results demonstrate that explainable machine learning can provide actionable insights by identifying stress-specific regions within crop images, thereby answering our hypothesis that early identification of drought stress using non-invasive imaging can improve decision-making in agricultural practices.

The novel integration of gradient-based visualization offers significant advances in model transparency, allowing agricultural practitioners to trust better and interpret the artificial intelligence-based system’s outputs. This interpretability is crucial for practical adoption in real-world agricultural settings, where understanding the basis of the model’s predictions is as important as accuracy. This framework offers a promising tool for improving crop management, water use efficiency, and overall sustainability by enabling targeted interventions such as precision irrigation.

While the framework has demonstrated strong performance, its application to other crops and environmental stress factors remains to be explored. Future efforts should expand its applicability, improve real-time processing capabilities, and address scalability across diverse agricultural conditions. This work presents an innovative, light-weight, and explainable approach to crop stress detection that has the potential to reshape current agricultural practices, ultimately fostering more sustainable and efficient crop management strategies.

## Data Availability

The original contributions presented in the study are included in the article/supplementary material. Further inquiries can be directed to the corresponding author.
